# Lung Oxidative Stress, DNA Damage, Apoptosis, and Fibrosis in Adenine-Induced Chronic Kidney Disease in Mice

**DOI:** 10.3389/fphys.2017.00896

**Published:** 2017-11-23

**Authors:** Abderrahim Nemmar, Turan Karaca, Sumaya Beegam, Priya Yuvaraju, Javed Yasin, Badreldin H. Ali

**Affiliations:** ^1^Department of Physiology, College of Medicine and Health Sciences, United Arab Emirates University, Al Ain, United Arab Emirates; ^2^Department of Histology and Embryology, Faculty of Medicine, Trakya University, Edirne, Turkey; ^3^Department of Internal Medicine, College of Medicine and Health Sciences, United Arab Emirates University, Al Ain, United Arab Emirates; ^4^Department of Pharmacology and Clinical Pharmacy, College of Medicine & Health Sciences, Sultan Qaboos University, Muscat, Oman

**Keywords:** adenine, chronic renal disease, lung, oxidative stress, apoptosis, DNA damage, Nrf2

## Abstract

It is well-established that there is a crosstalk between the lung and the kidney, and several studies have reported association between chronic kidney disease (CKD) and pulmonary pathophysiological changes. Experimentally, CKD can be caused in mice by dietary intake of adenine. Nevertheless, the consequence of such intervention on the lung received only scant attention. Here, we assessed the pulmonary effects of adenine (0.2% w/w in feed for 4 weeks)-induced CKD in mice by assessing various physiological histological and biochemical endpoints. Adenine treatment induced a significant increase in urine output, urea and creatinine concentrations, and it decreased the body weight and creatinine clearance. It also increased proteinuria and the urinary levels of kidney injury molecule-1 and neutrophil gelatinase-associated lipocalin. Compared with control group, the histopathological evaluation of lungs from adenine-treated mice showed polymorphonuclear leukocytes infiltration in alveolar and bronchial walls, injury, and fibrosis. Moreover, adenine caused a significant increase in lung lipid peroxidation and reactive oxygen species and decreased the antioxidant catalase. Adenine also induced DNA damage assessed by COMET assay. Similarly, adenine caused apoptosis in the lung characterized by a significant increase of cleaved caspase-3. Moreover, adenine induced a significant increase in the expression of nuclear factor erythroid 2–related factor 2 (Nrf2) in the lung. We conclude that administration of adenine in mice induced CKD is accompanied by lung oxidative stress, DNA damage, apoptosis, and Nrf2 expression and fibrosis.

## Introduction

The burden of chronic renal disease (CKD) has considerably increased globally reaching epidemic magnitudes of ~10% of certain populations (Stenvinkel, 2010). The occurrence and development of CKD is affected by numerous factors (e.g., diabetes, hypertension, and obesity) and is frequently accompanied by extra-renal manifestations such as pulmonary inflammation and edema, respiratory failure, sepsis, and heart failure (Domenech et al., 2017).

It is well-documented that there is interplay between the lung and the kidney, and several studies have reported association between CKD and pulmonary pathophysiological changes (Domenech et al., 2017). This communication happens owing to the increase in systemic inflammation, oxidative stress, and cause pulmonary injury (Domenech et al., 2017).

Animal models of CKD are important and much needed to improve our understanding of the pathophysiological changes seen in CKD and to implement new therapeutic strategies (Ali et al., 2010, 2013). A non-surgical model of CKD developed by the administration of adenine in diet has been shown to be a valid and reliable model to induce CKD in mice and rats (Yokozawa et al., 1986; Ali et al., 2010, 2013). Once adenine is consumed by the rats or mice, it is metabolized to 2,8-dihydroxyadenine, which precipitates and produces tubular crystals which subsequently damage the renal tissue (Yokozawa et al., 1986; Ali et al., 2010, 2013). While the effect of adenine on the development of CKD has been widely investigated, little is known on the extra-renal impact of such model of CKD. Recently, it has been reported that adenine induce CKD and cardiovascular damage in rats including increased ventricular fibrosis, systolic blood pressure and left ventricular stiffness, left ventricular diastolic dysfunction independent of the presence of hypertension and impaired vascular responses (Diwan et al., 2013; Ali et al., 2014a; Nakano et al., 2016). Since it has been reported that CKD is associated with effects on distant organs, including the lungs, in the present study, we wanted to test the hypothesis of the possible occurrence of pulmonary injury in animal model of CKD induced by adenine. Such interaction has, as far as we are aware, not been investigated. Therefore, the aim of this study is to assess the impact of adenine-induced CRF on lung histology, oxidative stress, DNA damage, apoptosis, and Nrf2 expression.

## Materials and methods

### Animals

Male TO mice (25–30 g, HsdOla:TO, Harlan, UK) were housed in light (12-h light:12-h dark cycle), relative humidity of 50–60% and temperature-controlled (22 ± 1°C) rooms. They had free access to commercial laboratory chow and filtered and UV-treated drinking water (Harmsco Hurricane Filtration Systems, Florida, USA). They were randomly divided into two groups and housed in cages, except 24 h before sacrifice, where they were housed individually in metabolic cages, to facilitate urine collection. The mice were weighed at the beginning of the experiment and just before sacrifice. Mice were cared for under a protocol approved by the Animal Research Ethics Committee of our college, and according to the NIH Guide for the Care and Use of Laboratory Animals, NIH publication no. 85-23, 1985. A total number of 62 mice were used to assess various biochemical, histological, and physiological parameters.

### Treatments

To induce CKD, mice received powdered diet containing adenine 0.2% w/w (i.e., 0.2 g adenine/100 g feed) for 4 weeks. This dose of adenine has been selected based on previous publications which have reported its efficacy in inducing CKD in mice (Ali et al., 2013, 2014b). Control animals received normal food for the same period of time. On day 28, mice were placed in metabolic cage and urine was collected over a 24-h period and the volume measured. On day 29, various renal and pulmonary endpoints were measured.

### Blood collection, histology, and biochemical analysis

Mice (*n* = 6 per group) were anesthetized with sodium pentobarbital (60 mg/kg, i.p.), and blood was drawn from the inferior vena cava in ethylenediaminetetraacetic acid (4%). The collected blood was centrifuged at 4°C for 15 min at 900 × g, and the plasma samples were stored at −80°C pending analysis.

The animals were sacrificed with an overdose of anesthesia. The lungs were fixed with 10 g/100 ml phosphate buffered formalin, excised, washed with ice-cold saline, blotted with filter paper, and weighed. Each lung was sectioned, put in a cassette, and dehydrated in increasing concentrations of ethanol, cleared with xylene, and embedded in paraffin. Sections of 3 μm were prepared from paraffin blocks and stained with hematoxylin and eosin or Masson's trichrome or Sirius red. The histological analyses were performed on five sections from each animal at 400x magnification in at least 10 different regions for each section. Histopathological evaluation was performed semi-quantatively: 0 was defined as normal lung, and 1, 2, 3, 4, and 5 were defined as the presence of inflammation involving 10, 10–30, 30–50, 50–80, or >80% of the lungs, respectively (Zhao et al., 2015).

Lung sections were stained with Masson's trichrome and Sirius red for collagen accumulation. Lung fibrosis was scored as following: 0: normal lung; grade 1: minimal fibrous thickening of alveolar or bronchial walls; grade 2: moderate thickening of walls to lung alveoli; grade 3: intensive fibrous thickening of alveolar or bronchial walls; grade 4: very intensive fibrous thickening of alveolar or bronchial walls (Chilakapati et al., 2015).

The concentrations of urea and proteins in plasma and creatinine in plasma and urine were spectrophotometrically measured using commercial kits (Roche Diagnostics, Indianapolis, IN, USA).

Commercially available ELISA kits were used to measure the urinary concentrations of kidney injury molecule-1 (KIM-1) (R & D systems, MN, USA) and neutrophil gelatinase-associated lipocalin (NGAL) activity (R & D systems, MN, USA).

### Measurement of lipid peroxidation (LPO), reactive oxygen species (ROS) and catalase levels in lung homogenates

In separate animals (*n* = 8 per group), mice were sacrificed by an overdose of sodium pentobarbital, and their lungs were quickly collected and rinsed with ice-cold PBS (pH 7.4) before homogenization, as described before (Nemmar et al., 2015). The homogenates were centrifuged for 10 min at 3,000 × g to remove cellular debris, and the supernatants were used for further analysis (Nemmar et al., 2015). Protein content was measured by Bradford's method. ROS were measured in whole lung tissue homogenates, which were obtained as described above, using 2′,7′-dichlorofluorescein diacetate (Molecular Probes, Eugene, OR, USA) as a fluorescent probe as previously described (LeBel et al., 1990; Nemmar et al., 2013b). The results were normalized as ROS produced per milligram of protein.

NADPH-dependent membrane lipid peroxidation was measured as thiobarbituric acid reactive substance using malonedialdehyde as standard (Sigma-Aldrich Fine Chemicals, St Louis, MO, USA) (Nemmar et al., 2012a).

Measurement of catalase activity was carried out in control and adenine-treated mice using spectrophotometric method with commercially-available kits (Cayman Chemical, Ann Arbor, MI, USA).

### DNA damage assessment by comet assay

Promptly after sacrifice, the lungs from control and adenine-treated mice (*n* = 5 per group) were removed, and used for the assessment of DNA damage using the COMET assay as performed as described before (Olive et al., 1994; de Souza et al., 2014), and the assessment of length of the DNA migration (i.e., diameter of the nucleus plus migrated DNA) was measured using image analysis Axiovision 3.1 software (Carl Zeiss, Canada) (Hartmann and Speit, 1997; Nemmar et al., 2016b, 2017b).

### Western blot analysis

Protein expressions for cleaved caspase-3 and Nrf2 were measured using Western blotting techniques. Lung tissues harvested from the mice (*n* = 4 per group) were snap frozen immediately with liquid nitrogen and stored at −80°C. Later, the tissues were weighed, rinsed with saline and homogenized with lysis buffer (pH 7.4) containing NaCl (140 mM), KCl (300 mM), trizma base (10 mM), EDTA (1 mM), Triton X-100 0.5/100 ml distilled water, sodium deoxycholate 0.5 g/100 ml distilled water, protease and phosphatase inhibitor. The homogenates were centrifuged for 20 min at 4°C. The supernatants were collected and protein estimation was made using a Pierce bicinchoninic acid protein assay kit (Thermo Scientific). For cleaved caspase-3, a sample of protein (80 μg) was electrophoretically separated by 12% sodium dodecyl sulfate polyacrylamide gel electrophoresis and then transferred onto polyvinylidene difluoride membranes. For Nrf2, a sample of protein (70 μg) was electrophoretically separated by 10% sodium dodecyl sulfate polyacrylamide gel electrophoresis and then transferred onto polyvinylidene difluoride membranes. The immunoblots were then blocked with 5% non-fat milk and subsequently probed with either the rabbit monoclonal cleaved caspase-3 antibody (1:250 dilution, Cell Signaling Technology) or rabbit monoclonal Nrf2 antibody (1:2,000 dilution, Abcam) at 4°C overnight. The blots were then incubated with goat anti-rabbit IgG horseradish peroxidase conjugated secondary antibody (1:5,000 dilution, Abcam) for 2 h at room temperature and developed using Pierce enhanced chemiluminescent plus Western blotting substrate Kit (Thermo Scientific). The densitometric analysis of the protein bands was performed with Typhoon FLA 9500 (GE Healthcare Bio-Sciences AB, Uppsala, Sweden). Blots were then re-probed with mouse monoclonal GAPDH antibody (1:5,000 dilution, Abcam) and used as a control.

### Airway reactivity to methacholine

At the end of the treatment period (day 29), airway hyperreactivity responses were measured in control and adenine-treated mice using a forced oscillation technique (FlexiVent, SCIREQ, Montreal, Canada). Airway resistance (R) was assessed after increasing exposures to methacholine. Mice (*n* = 8 in each group) were anesthetized with an intraperitoneal injection of pentobarbital (70 mg/kg). The trachea was exposed and an 18-gauge metal needle was inserted into the trachea. Mice were connected to a computer-controlled small animal ventilator and quasi-sinusoidally ventilated with a tidal volume of 10 ml/kg at a frequency of 150 breaths/min and a positive end-expiratory pressure of 2 cm H_2_O to achieve a mean lung volume close to that during spontaneous breathing. After measurement of a baseline, each mouse was challenged with methacholine aerosol, generated with an in-line nebulizer and administered directly through the ventilator for 5 s, with increasing concentrations (0, 0.625, 2.5, 10, and 40 mg/ml). R was measured using a “snapshot” protocol each 20 s for 2 min. The mean of these five values was used for each methacholine concentration, unless the coefficient of determination of a measurement was smaller than 0.95. For each mouse, R was plotted against methacholine concentration (from 0 to 40 mg/ml) (Nemmar et al., 2012b, 2013a).

### Statistics

All statistical analyses were performed with GraphPad Prism Software version 7.03. Shapiro–Wilk normality test was applied, and the data was found normally distributed. Therefore, comparisons between groups were performed by unpaired student's *t*-test. The data in figures were reported as mean ± SEM. *P* < 0.05 are considered significant.

## Results

### Physiological and biochemical data

Table 1 presents some physiological and biochemical parameters in plasma of control mice and those which received adenine administration in the feed (0.2% w/w, for 4 weeks). As expected, compared with control group, adenine treatment induced a significant increase in urine output (*P* < 0.001), urea (*P* < 0.001), and creatinine (*P* < 0.001) concentrations, and it decreased the body weight (*P* < 0.001) and creatinine clearance (*P* < 0.05).

**Table 1:** Some physiological and biochemical parameters in and urine of control mice and those which received adenine administration in the feed (0.2% w/w, for 4 weeks).

**Parameters/Group**	**Control**	**Adenine**
Urine output (ml/24 h)	3.0 ± 0.4	10.3 ± 1.2^**^
Change in body weight (%)	3.2 ± 2.2	−12.3 ± 1.2^**^
Urea (μmol/l)	6.1 ± 0.5	18.2 ± 1.3^**^
Creatinine (μmol/l)	14.5 ± 0.7	22.0 ± 1.3^**^
Creatinine clearance (ml/min)	0.2 ± 0.04	0.09 ± 0.008^*^

*Values in the table are mean ± SEM (n = 6). Adenine was added to the feed at a concentration of 0.2% w/w, for 4 weeks*.

* P < 0.05 and

** *P < 0.001 (control vs. adenine group)*.

**Figure 1:**
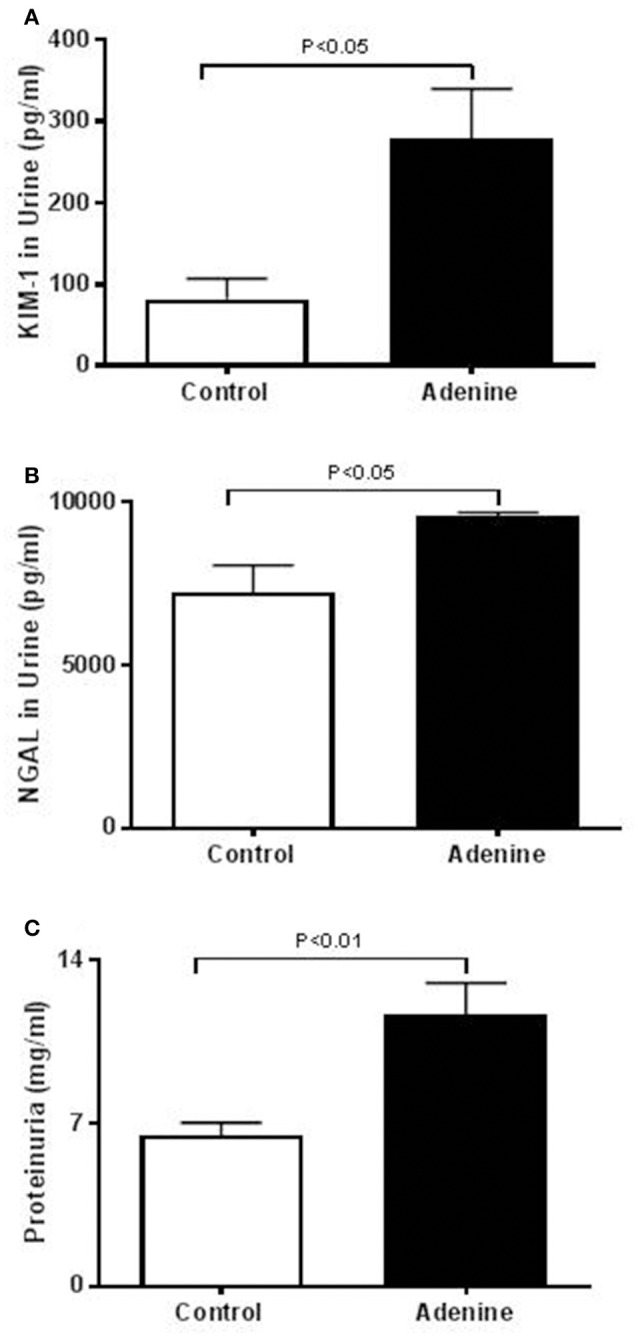
Urinary concentrations of kidney injury molecule-1 (KIM-1, **A**), neutrophil gelatinase-associated lipocalin (NGAL, **B**), and proteins **(C)** in control mice and those treated with adenine mixed in the feed (0.2% w/w, for 4 weeks). Mean ± SEM (*n* = 6 in each group).

The urinary concentrations of proteins, KIM-1, and NGAL were significantly increased in adenine-treated group compared with the control one (Figure 1).

### Lung histology

**Figure 2:**
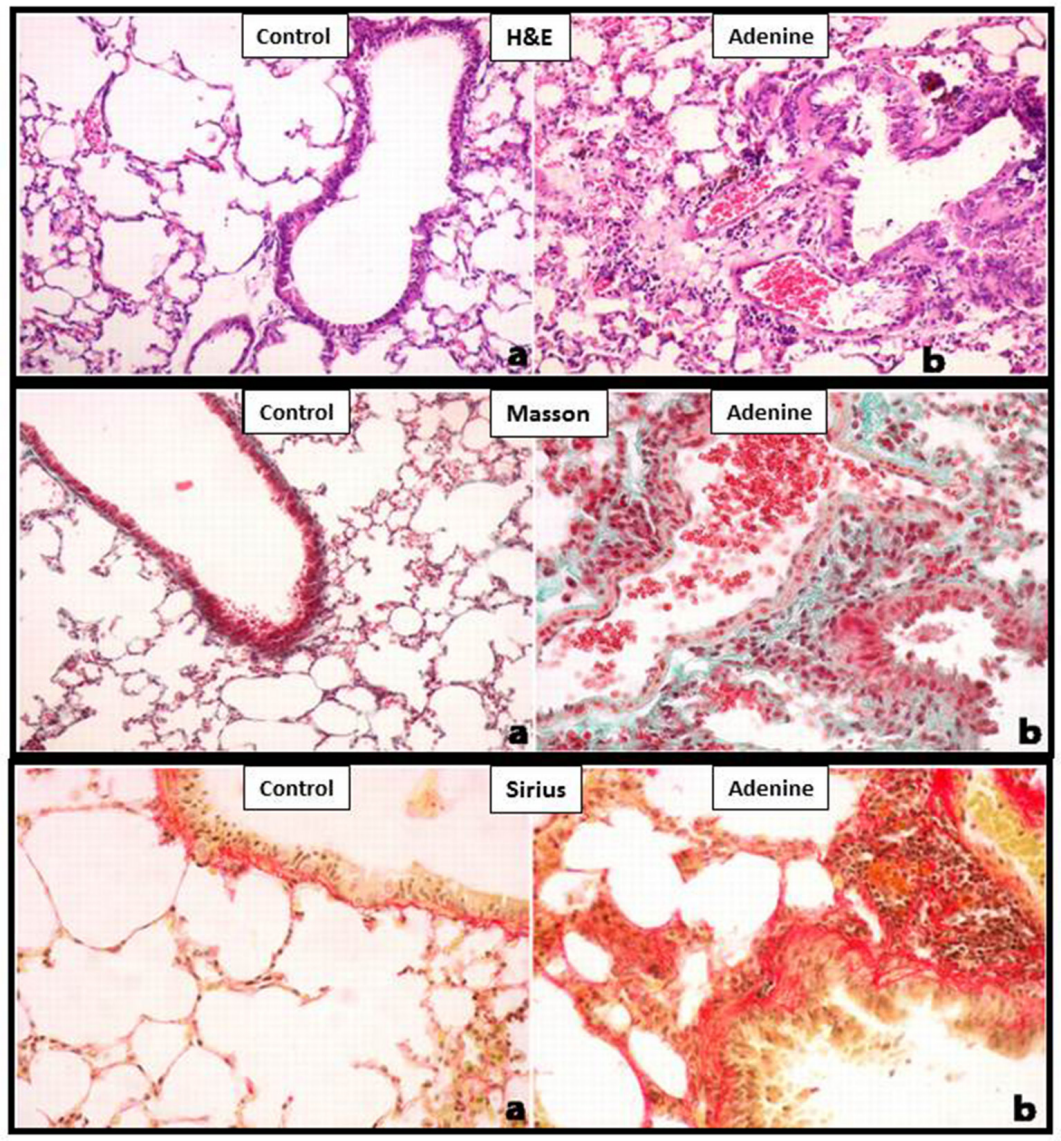
Representative light microscopy sections of lung tissues of control mice and those treated with adenine mixed in the feed (0.2% w/w, for 4 weeks), stained with H&E or Masson's trichrome or Sirius red. All magnifications: 400X. The control group shows normal lung architecture and histology. Adenine-treated group shows polymorphonuclear leukocyte infiltration in alveolar and bronchial walls, injury, and fibrosis. See this figure for statistical analysis of scores of lung injury and fibrosis.

**Figure 3:**
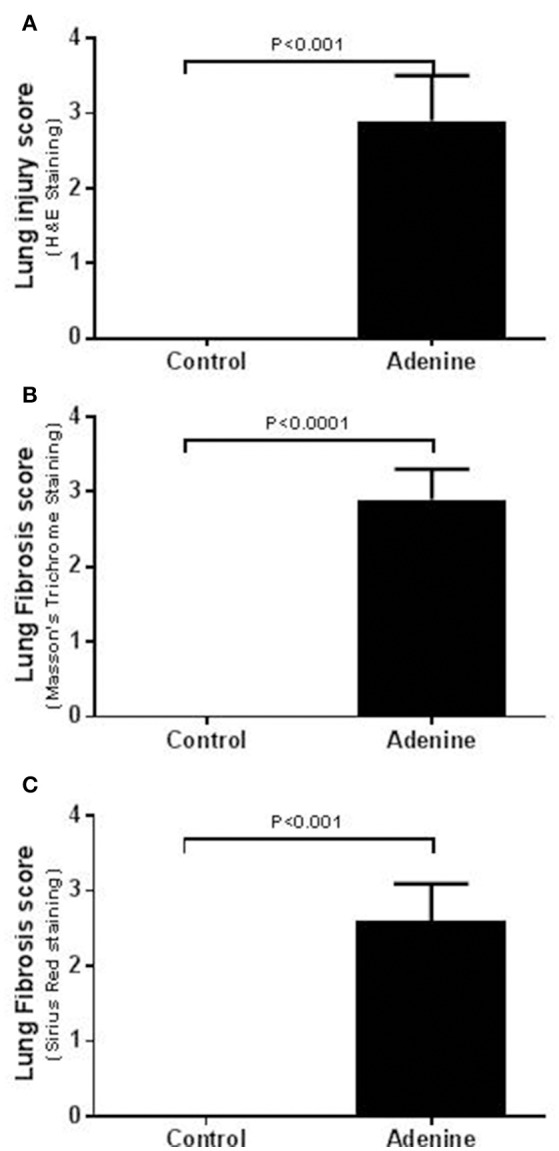
Lung injury scores in tissues stained with H&E **(A)** and lung fibrosis scores after Masson's **(B)** or Sirius red **(C)** staining in control mice and those treated with adenine mixed in the feed (0.2% w/w, for 4 weeks). Mean ± SEM (*n* = 6 in each group).

Light microscopy analysis of the lung sections stained with H&E obtained from control mice exhibited normal structure (Figure 2). Compared with control group, histological evaluation (Figure 2), and scoring (Figure 3) showed that adenine treatment induced polymorphonuclear leukocyte infiltration in alveolar and bronchial walls, injury, and fibrosis (Figure 2). Moreover, the scores for lung injury and fibrosis were significantly increased in adenine-treated group compared with control mice (Figure 3).

### LPO, ROS, and catalase levels in lung homogenates

**Figure 4:**
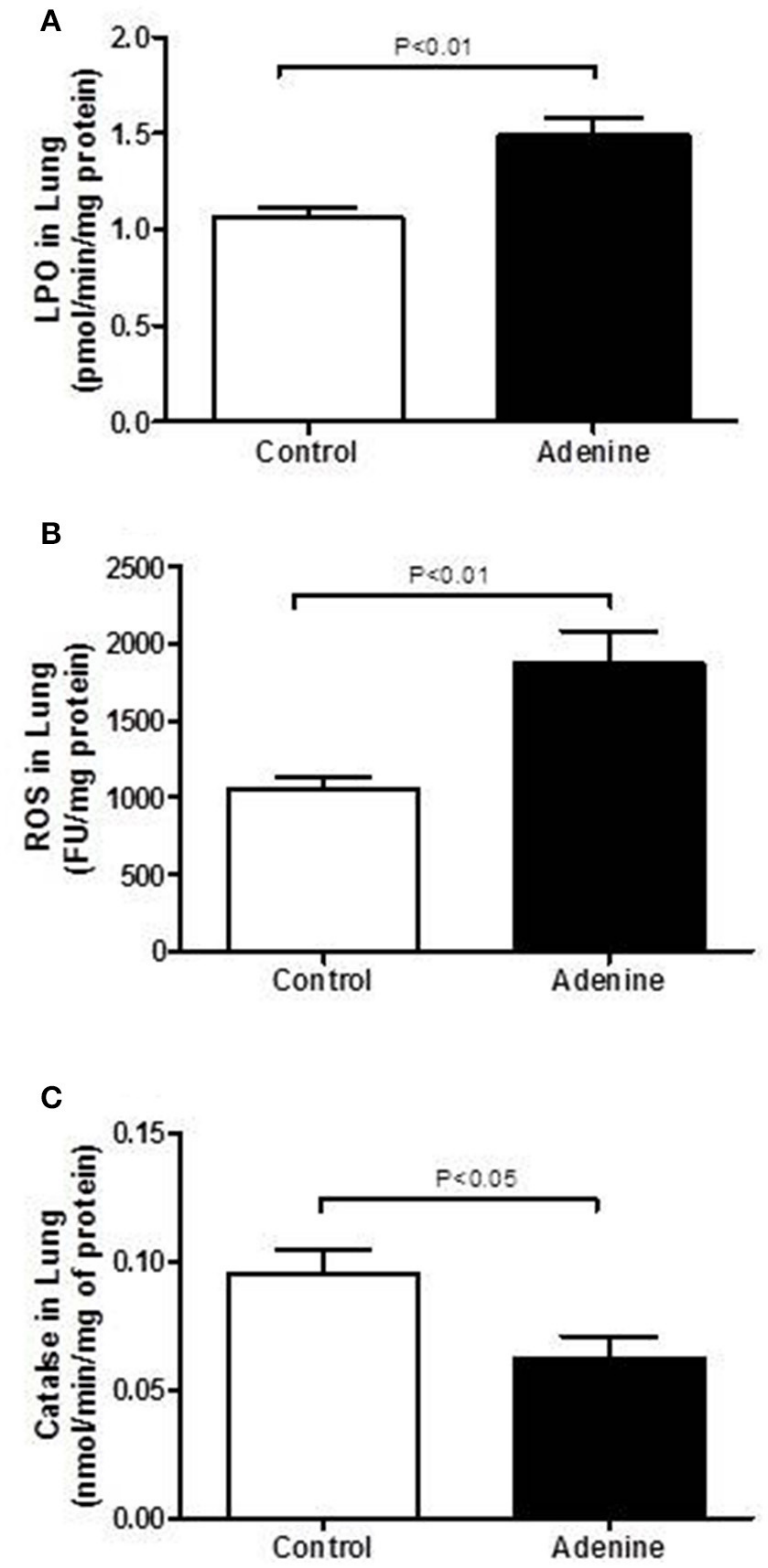
Lung levels of lipid peroxidation (LPO, **A**), reactive oxygen species (ROS, **B**) and catalase **(C)** in control mice and those treated with adenine mixed in the feed (0.2% w/w, for 4 weeks). Mean ± SEM (*n* = 8 in each group).

Figure 4 illustrates the effects of adenine treatment on markers of oxidative stress including LPO, ROS, and catalase in lung homogenates. Compared with control group, adenine administration induced a significant increase of LPO concentrations in lung homogenates (*P* < 0.01; Figure 4A). Likewise, ROS levels were significantly increased in lung homogenate of adenine-treated mice (*P* < 0.01; Figure 4B) compared with control group. Figure 4C shows that compared with control group, adenine caused a significant decline in the activity of the antioxidant catalase (*P* < 0.05).

### Lung DNA damage

**Figure 5:**
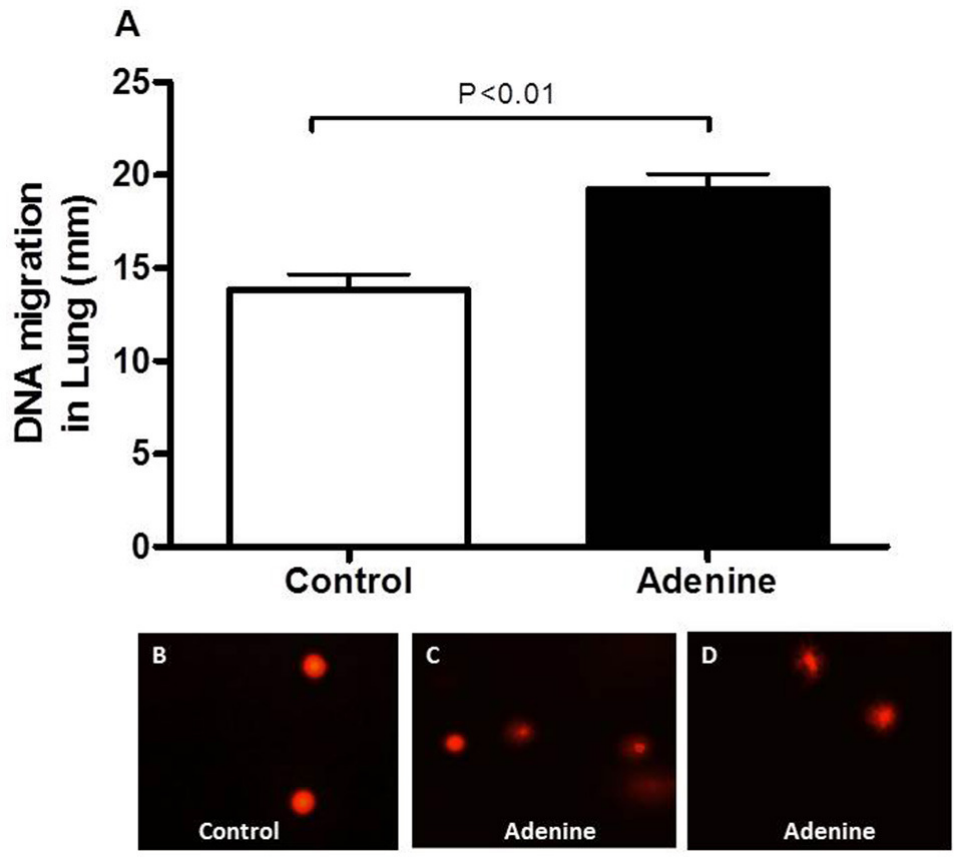
DNA migration (mm) in the lung tissues assessed by Comet assay in control mice and those treated with adenine mixed in the feed (0.2% w/w, for 4 weeks) **(A)**. Data are means ± SEM (*n* = 5). Representative images illustrating the quantification of the DNA migration by the Comet assay under alkaline conditions, in control **(B)** and adenine-treated mice **(C,D)**.

Figure 5 shows the effect of adenine administration on lung DNA damage assessed by COMET assay. Adenine caused a significant DNA migration in the lung (*P* < 0.01) compared with control group.

### Western blot analysis for the detection of caspase-3 and Nrf2

**Figure 6:**
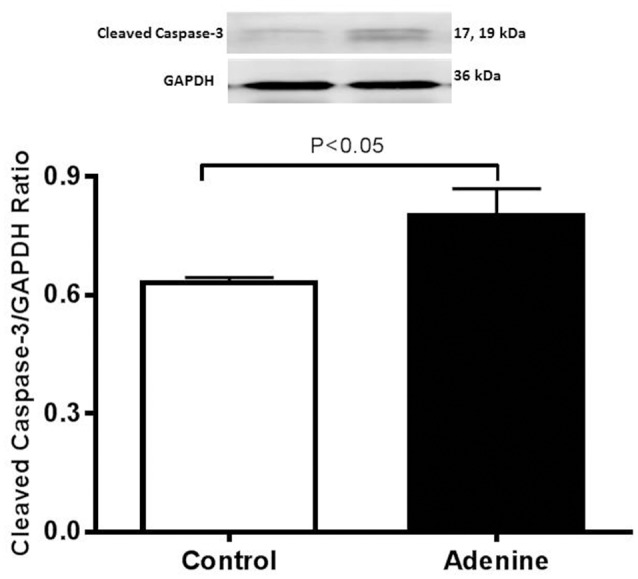
Western blot analysis and graphic representation of cleaved caspase-3 protein levels in the lung tissues in control mice and those treated with adenine mixed in the feed (0.2% w/w, for 4 weeks). Data are mean ± SEM (*n* = 4 in each group).

**Figure 7:**
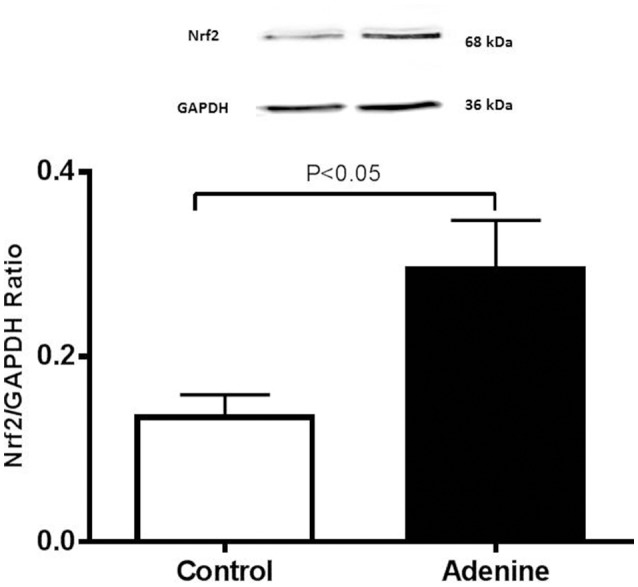
Western blot analysis and graphic representation of nuclear factor erythroid 2-related factor 2 (Nrf2) protein levels in the lung tissues in control mice and those treated with adenine mixed in the feed (0.2% w/w, for 4 weeks). Data are mean ± SEM (*n* = 4 in each group).

Western blots for the detection of cleaved caspase-3 and Nrf2 are represented in Figures 6 and 7 and Supplementary Figure 1.

Figure 6 illustrates the effect of adenine on apoptotic marker cleaved caspase-3. Compared with control group, adenine treatment induced a significant increase of cleaved caspase-3 (*P* < 0.05).

Figure 7 shows that compared with the control group, the treatment of mice with adenine induced a significant increase in the expression of Nrf2 (*P* < 0.05).

### Airway hyperreactivity to methacholine

**Figure 8:**
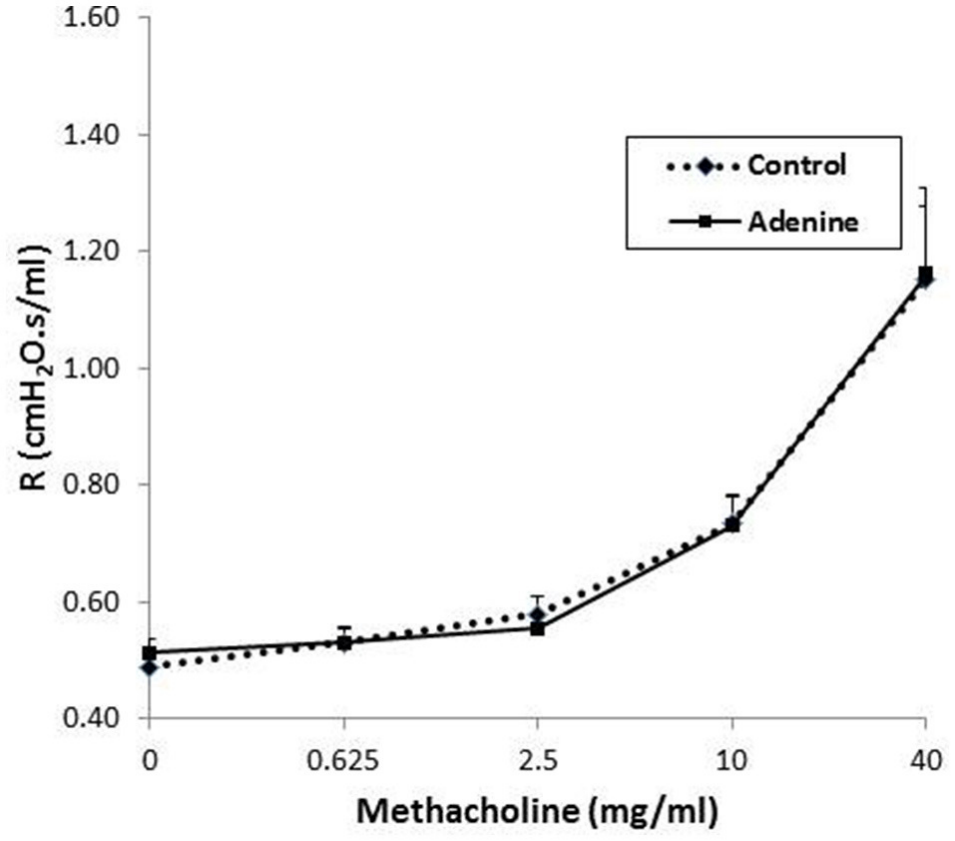
Airway hyper-responsiveness. The airway resistance (*R*), after increasing concentrations of methacholine (0–40 mg/ml), was measured via the forced oscillation technique (FlexiVent) in control mice and those treated with adenine mixed in the feed (0.2% w/w, for 4 weeks). Data are mean ± SEM (*n* = 8).

The airway hyperreactivity to methacholine (0–40 mg/ml), measured by the forced oscillations technique, in control mice and those administered with adenine is presented in Figure 8. No differences in airway resistance has been observed between the control and adenine-treated groups following the administration of various concentrations of methacholine.

## Discussion

In this work, we showed that adenine-induced CKD in mice caused histopathological, biochemical, and molecular pulmonary changes, including injury, fibrosis, oxidative stress, DNA damage, apoptosis, and expression of the transcription factor Nrf2.

Lung and kidney functions are closely linked in both health and disease (Visconti et al., 2016; Domenech et al., 2017). It is well-established that renal disease can adversely affect the lungs and vice versa. In fact, the lungs could be affected by inflammation and oxidative stress related to a primary kidney disease (Visconti et al., 2016; Domenech et al., 2017). Conversely, kidney function could be altered by injury taking place in the lungs causing the release of mediators into the lung, which may subsequently pass into the systemic circulation and cause kidney dysfunction (Visconti et al., 2016; Domenech et al., 2017).

Animal models of CKD are essential for answering mechanistic questions related to the diseases and for detection of toxicities and developing preventive and/or therapeutic strategies aiming mitigating/preventing kidney injury (Susztak et al., 2008). The most frequently used animal model for progressive renal failure is the remnant kidney of 5/6 nephrectomized rat in which there are decreased renal mass and nephron number, and is caused by infarction or surgical removal of both poles, with deletion of the contralateral kidney (Kujal and Vernerova, 2008). The other model of CKD is induced by the administration of adenine mixed with the feed at a concentration of 0.75% in rats or 0.2% in mice, w/w, for 4 weeks (Yokozawa et al., 1986; Ali et al., 2013, 2015). We have recently used this model and demonstrated that intratracheal instillation of diesel exhaust particles aggravate renal oxidative stress, inflammation, and DNA damage in mice with adenine-induced CKD (Nemmar et al., 2016a). We and others have also used this model to show the occurrence of gastrointestinal or cardiovascular complications after the induction of CKD with adenine (Ali et al., 2013, 2014b; Diwan et al., 2013; Nakano et al., 2016). However, as far as we are aware, no study to date has investigated the *in vivo* pulmonary consequence of this valid and well-established model of CKD.

As reported before (Ali et al., 2013; Nemmar et al., 2016a), in this study, we confirmed that adenine feeding (0.2% for 4 weeks) caused a significant decrease in urine output, urea and creatinine concentrations, and it decreased the body weight and creatinine clearance. Moreover, along with the proteinuria, we also showed that the urinary concentrations of KIM-1 and NGAL which are both sensitive biomarkers of renal injury (Waring and Moonie, 2011) were significantly increased in adenine-treated group. It is well-established in mice that treatment with adenine induce kidney injury and fibrosis (Ali et al., 2013; Nemmar et al., 2016a) but its *in vivo* effect on the lung has not been reported before. Our data show that treatment with adenine in mice induced polymorphonuclear leukocyte infiltration in alveolar and bronchial walls, lung injury, and fibrosis. Such effect has not been reported before. Using an animal model of acute renal failure induced by a single intraperitoneal injection of cisplatin (6 mg/kg) in rats, it has been shown that cisplatin increased cellularity of interalveolar interstitium and induced mild congestion (Nemmar et al., 2010).

Oxidative stress is a consequence of an imbalance between formation and removal of ROS (Birben et al., 2012). Once generated in excess, free radicals and oxidants induce oxidative stress which damages cell membranes and other structures including proteins, lipids, lipoproteins, and DNA (Birben et al., 2012). In order to delineate the mechanisms underlying the pulmonary injurious effect of adenine on the lung, we have measured markers of oxidative stress including LPO, ROS and the antioxidant enzyme catalase in lung homogenates. Our data show that adenine treatment caused a significant increase in LPO and ROS and decreased in catalase activity which indicates that this antioxidant has been consumed as a result of oxidative stress (Nemmar et al., 2017a). We have recently reported an increase of LPO and ROS and a decrease of catalase in kidney homogenates of mice treated with adenine (Nemmar et al., 2016a). Moreover, we have also showed that concomitant administration of adenine and diesel exhaust particles aggravated these markers of oxidative stress in kidney homogenates (Nemmar et al., 2016a). One of the limitations of the results of the antioxidant catalase measured in this work relates to the specificity of the conventional assay that we used. Despite being widely used, and unlike novel techniques such as liquid chromatography-mass spectrometry, the assay used does not differentiate totally between activity of catalase and that of the other peroxidases (Cipak et al., 2017). Nonetheless, our results were comparing the activity of this antioxidant index in the controls and adenine-treated mice. Invariably, we have found that this antioxidant was significantly lower in the adenine-treated mice. This confirms the data previously reported by us and by several others using similar assay (Kadowaki et al., 2012; Palabiyik et al., 2013; Ali et al., 2016; Hussein et al., 2016).

Our present data show that adenine treatment induces DNA damage in the lung. This result suggests that adenine administration caused this effect on DNA by prompting an environment of oxidative stress. It has been reported that DNA damage induces apoptosis in a DNA replication dependent way by activating the mitochondrial damage pathway in fibroblasts (Kaina, 2003). Caspases are key mediators of apopotosis (Savitskaya and Onishchenko, 2015). It has been reported that ROS are able to regulate apoptosis characteristically via caspase-3 activation (Savitskaya and Onishchenko, 2015). Here, we show that adenine induced a significant increase in cleaved caspase-3 expression. The occurrence of DNA damage and apoptosis in kidney of mice and rat treated with adenine has been reported before (Shuvy et al., 2011; Ali et al., 2013; Diwan et al., 2013).

Nrf2 is a transcription factor which plays an important role in the instigation of antioxidant enzymes to respond to oxidative stress (Barancik et al., 2016). Several pharmacologic approaches involve the induction of Nrf2 antioxidant response to protect against oxidative stress (Barancik et al., 2016). Our data show a significant increase in the expression of Nrf2 in lung homogenate of mice treated with adenine suggesting that adenine treatment could trigger adaptive responses that counterbalance the potentially damaging activity of oxygen radicals induced by adenine administration. Our data corroborate the finding of a recent study which showed that Nrf2 expression is increased in lipopolysaccharide (LPS)-induced lung injury in mice (Zhu et al., 2017). In the same study, it was shown that geraniin, an ellagitannin isolated from *Phyllanthusurinaria Linnnatural* which has anti-inflammatory and antioxidant actions, inhibited NF-κB activation and induced an up-regulation of Nrf2 expression that resulted in the attenuated LPS-induced acute lung injury (Zhu et al., 2017).

Our data demonstrate that the assessment of airway hyperreactivity to methacholine measured by the forced oscillations technique, showed no difference in airway resistance between the control and adenine-treated groups. Similarly, a recent study reported the development of a mouse model of chronic idiopathic pulmonary fibrosis induced by belomycin administration, showed the occurrence of lung injury and fibrosis but with no change in airway resistance (Limjunyawong et al., 2014).

Taken together, our data show, for the first time, the *in vivo* pulmonary impact of adenine-induced kidney disease, and add to the existing knowledge on the extra-renal effect of such model of CKD including the cardiovascular and gastrointestinal systems (Ali et al., 2013, 2014a,b; Diwan et al., 2013). However, and surprisingly, a recent study found that adenine-induced kidney disease in rats attenuates ischemia-reperfusion injury in an *ex-vivo* isolated perfused rat lung model (Peng et al., 2017). The reason for this discrepancy cannot be readily explained but it could be explained by the fact that our study in mice is an *in vivo* one and reflects better the interaction between the lung and the kidney whereas that of Peng et al. (2017) is an *ex vivo* study that uses an isolated perfused rat lung model. Moreover, clinical studies have prominently reported that CKD can adversely affect the lungs and vice versa (Visconti et al., 2016; Domenech et al., 2017).

We conclude that the administration of adenine in mice induced CKD and also caused lung injury including fibrosis, oxidative stress, DNA damage, apoptosis, and expression of Nrf2. Additional studies are required to quantify the expression of heme oxygenase-1 and the concentrations of adenine in the lung and kidney, and to assess whether the effect observed in the lung is an indirect consequence of CKD induced by adenine or a direct effect of adenine on the lung or the combination of the two processes.

## Ethics statement

This project was reviewed and approved by our Institutional Review Board of the United Arab Emirates University, College of Medicine and Health Sciences, and experiments were performed in accordance with protocols approved by the Institutional Animal Care and Research Advisory Committee.

## Author contributions

All authors have read and approved the manuscript. AN designed, planned, supervised the experiments and wrote the article. TK performed the histology part of the work. SB, PY, and JY performed the experiments. BA contributed in the design of the study and the writing of the manuscript.

### Conflict of interest statement

The authors declare that the research was conducted in the absence of any commercial or financial relationships that could be construed as a potential conflict of interest.
