# Supplementary figures and images for: Lung Oxidative Stress, DNA Damage, Apoptosis, and Fibrosis in Adenine-Induced Chronic Kidney Disease in Mice
Source: Front Physiol. 2017 Nov 23;8:896. doi: 10.3389/fphys.2017.00896 (PMC5703828; doi:10.3389/fphys.2017.00896)

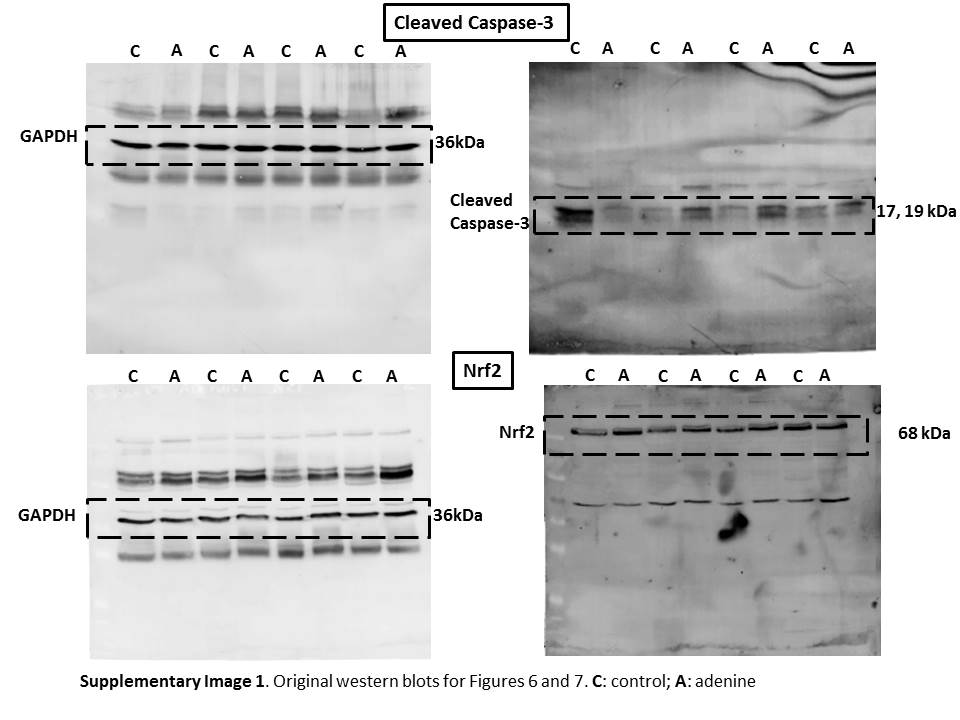

Supplement: Supplementary file 1 [file Image1.JPEG]
